# Development of a Framework for Evaluating Large Language Model Safety and Reliability: a Proof-of-Concept Evaluation

**DOI:** 10.1007/s10916-026-02459-1

**Published:** 2026-09-23

**Authors:** Fangyan Liu, Zhi Liu, Xiaolu Fei, Jingyu He, Jin Xing, Jia Li, Piu Chan

**Affiliations:** 1 Department of Emergency, National Clinical Research Center for Geriatric Diseases, Xuanwu Hospital of Capital Medical University, Beijing, 100053 China; 2 Department of Emergency, Xiongan Xuanwu Hospital, No. 1, Xili Road, Xiongan New Area, Hebei 070001 China; 3 Information Center, National Clinical Research Center for Geriatric Diseases, Xuanwu Hospital of Capital Medical University, Beijing, 100053 China; 4 Information Center, Xiongan Xuanwu Hospital, No. 1, Xili Road, Xiongan New Area, Hebei 070001 China; 5 Department of General Surgery, Xuanwu Hospital of Capital Medical University, No. 45, Changchun Street, Beijing, 100053 China; 6 Department of General Surgery, Xiongan Xuanwu Hospital, No. 1, Xili Road, Xiongan New Area, Hebei 070001 China; 7 National Clinical Research Center for Geriatric Diseases, Department of Geriatrics, Xuanwu Hospital of Capital Medical University, 45 Changchun Street, Beijing, 100053 China; 8 Beijing Institute of Geriatrics, Beijing, 100053 China; 9 Clinical Center for Parkinson’s Disease, Key Laboratory for Neurodegenerative Disease of the Ministry of Education, Beijing Key Laboratory for Parkinson’s Disease, Capital Medical University, Beijing, 100053 China

**Keywords:** Large language models, Clinical decision support systems, AI safety evaluation, Response consistency, AI governance, Health informatics

## Abstract

Large language models (LLMs) are entering clinical decision support faster than methodology can characterise their safety. Aggregate accuracy treats all errors as interchangeable and cannot support safe deployment under Software as a Medical Device (SaMD) and EU AI Act frameworks. To develop and demonstrate a framework for evaluating large language model safety and reliability using catastrophic-failure frequency and response reproducibility, incorporating a pre-specified error taxonomy, difficulty-stratified analysis, and two-layer response consistency. The framework was applied to 54 diagnostically challenging emergency cases from non-public institutional records. Six LLMs were accessed via application programming interfaces (APIs) (text-only, zero-shot, defaults; August–December 2025) and queried three times each, producing 972 physician-scored responses. Aggregate scores concealed heterogeneity: anchoring-bias failure (score ≤ 3) varied nearly four-fold (6.9%–26.4%) and rare-disease recognition six-fold (6.1%–37.9%); catastrophic (dangerous-recommendation, score ≤ 2) rates were two-tiered (1.5% for the safest two vs. 6.0% for the rest; *p* < 0.001), though within-tier ranks were inseparable (2–12 per model). Despite a middle-tier mean, GPT-5.1 was least reproducible (within-case SD 2.69) and fell within the higher catastrophic-failure tier; across models, 31 of 34 dangerous combinations were stochastic, not systematic—a failure mode hidden by aggregate metrics. Open-source DeepSeek R1 was statistically equivalent to Gemini 3 Pro within a 1.5-point margin by two one-sided tests (TOST: Δ − 0.09; 90% CI − 0.47 to 0.30; *p* < 0.001). In this single-centre proof-of-concept, aggregate accuracy was insufficient for safety characterisation: models with indistinguishable mean accuracy carried different catastrophic-failure tiers and reproducibility profiles. Error-type and response-consistency profiling may inform model selection, ensemble design, and conformity assessment.

**Supplementary Information:**

The online version contains supplementary material available at https://doi.org/10.1007/s10916-026-02459-1.

## Introduction

Large language models (LLMs) are being deployed into high-stakes decision-making domains faster than evaluation methodology can characterise their safety-relevant failure modes [1]. Current paradigms predominantly rely on aggregate accuracy on standardised benchmarks [2–6], an approach that treats all errors as interchangeable. In safety-critical applications errors are not equivalent: an anchoring-driven misdiagnosis carries different consequences from a rare disease oversight, and unpredictable failure poses different deployment risks from consistent failure [7, 8]. Whether LLMs with comparable aggregate performance exhibit equivalent safety profiles—or whether aggregate accuracy masks divergent vulnerability patterns—remains largely uncharacterised.

This marks a discontinuity in how decision support is built and evaluated. First-generation clinical decision support systems (CDSS) were knowledge-based (IF–THEN rules, probabilistic models) validated against deterministic, reproducible outputs [9, 10] their successors were narrow, task-specific machine-learning tools regulated for a single indication [7]. Generative LLMs differ on three axes the established toolkit was not designed for: they are general-purpose rather than indication-specific, their outputs are open-ended free text rather than a bounded label, and they are stochastic—the same query can yield differently safe recommendations on repeated runs. Regulatory frameworks distinguish lower-risk decision support a clinician can independently review from higher-risk Software as a Medical Device (SaMD) [8], a boundary open-ended output strains; yet evaluation has largely carried over aggregate-accuracy metrics that capture none of these properties.

Emergency medicine provides a demanding testbed. Emergency diagnosis involves undifferentiated presentations, incomplete information, and time-critical decisions—conditions where cognitive biases substantially contribute to clinician diagnostic error [11, 12]. Diagnostic accuracy for some rare conditions can fall below 50% [13, 14], and anchoring bias is among the most consequential sources of clinical error [15]. Recent work proposes that LLMs may inherit such biases yet also mitigate them through structured reasoning [16]; whether this holds across architectures is untested.

Four specific gaps limit current evaluation practice. First, studies overwhelmingly report aggregate performance without error-type characterisation, leaving clinically consequential failure patterns invisible to summary statistics [7, 8]. Second, response consistency is underexplored; only 15.1% of LLM medical evaluation studies report stochasticity-handling parameters [17], and semantically equivalent prompts produce significant clinical-LLM variability [18]. Third, high-performing open-source models such as DeepSeek R1 [19, 20] introduce a deployment dimension—on-premises feasibility under the Health Insurance Portability and Accountability Act (HIPAA) and the General Data Protection Regulation (GDPR) [21]—yet whether they match proprietary systems in safety profile, not just aggregate accuracy, remains uncharacterised. Fourth, most evaluations are Western and English-language [22]; cross-linguistic generalisability is incompletely characterised—a gap the present study begins to address in a Chinese tertiary-hospital setting.

We propose and illustrate a multi-dimensional safety-profiling framework (hereafter “the framework”) integrating three components: (1) a pre-specified error taxonomy of anchoring bias, rare disease oversight, and iatrogenic risk; (2) difficulty-stratified performance analysis; and (3) a two-layer response consistency assessment examining both round-level mean stability across repeated queries and case-level reproducibility on safety-critical presentations. Crucially, the primary safety endpoints are the frequency of catastrophic (dangerous) recommendations and response reproducibility, with mean performance retained only as a supporting analysis. The framework was applied to six frontier LLMs (open-source and proprietary; reinforcement-learning and supervised fine-tuning) across 54 diagnostically challenging emergency cases; two emergency medicine specialists independently scored 972 responses.

## Methods

### Study Design and Ethical Approval

This retrospective evaluation assessed six frontier LLMs on diagnostically challenging emergency cases from a Chinese tertiary hospital. The study was approved by the Institutional Review Board of Xuanwu Hospital, Capital Medical University (Protocol No. XA-KS2024-017-002); informed consent was waived as all clinical data were de-identified.

### Case Selection

Fifty-four cases were retrospectively selected from institutional records (2015–2024) by a panel of 28 emergency physicians across twelve clinical domains. Inclusion required (i) a confirmed final diagnosis, (ii) diagnostic complexity beyond pattern recognition, and (iii) absence from publicly available medical-examination or question-answer databases to minimise training-data contamination. Each physician submitted two to three candidate cases (73 total), reviewed in departmental case conferences; 19 were excluded (8 with insufficient clinical information, 5 with an unconfirmed final diagnosis, and 6 with duplicate themes), yielding 54. Of these, 53 had never appeared online, and the single exception (Case 20) did not drive results (Section 3.7). The case list, diagnoses, difficulty classifications, and full eligibility criteria are in Supplementary Methods and Table S2.

### LLM Selection and Query Protocol

Six LLMs spanning open-source and proprietary architectures were evaluated: DeepSeek R1, DeepSeek V3.1, Claude Sonnet 4.5, GPT-5.1, Grok 4, and Gemini 3 Pro (full specifications in Supplementary Table S3). All models were accessed via official APIs during August–December 2025 under default settings. Each case was queried three times per model (≥ 24 h apart, randomised order) using a standardised Chinese-language prompt (Supplementary Box S2), submitted as an independent API session to prevent cross-contamination. No system-role messages, few-shot examples, or chain-of-thought directives were used; a brief role definition embedded in the user-role prompt (Supplementary Box S2) was identical across all models and cases. All six are general-purpose models not fine-tuned for clinical use. They are not fully independent, as DeepSeek R1 and V3.1 share a base-model lineage (R1 a reasoning-oriented variant), so contrasts involving these two should be read with that in mind. All inputs were text-only (multimodal capabilities unused); each case was manually transcribed into a standardised text template (Supplementary Methods).

### Evaluation Framework

Two board-certified emergency physicians (F.L., Z.L.), blinded to model identity, independently scored each response across three dimensions—diagnosis, next-step recommendations, and treatment planning—each on a 1–5 integer scale (raters were not permitted to assign half-points) (total 3–15; rubric in Supplementary Table S4). Consensus scores were computed as the mean of the two raters.

### Error Taxonomy

Three error categories were pre-specified: rare disease recognition (prevalence < 1:10,000; 22 cases), anchoring bias susceptibility (misleading initial findings; 24 cases), and iatrogenic risk identification (treatment-complication recognition; 11 cases). Three cases (7, 18, 24) were classified under more than one category. Per the rubric (Supplementary Table S4), any dimension scoring ≤ 2 was classified as a dangerous (catastrophic) recommendation—potentially fatal, contraindicated, or grossly inadequate—and any dimension scoring ≤ 3 as a failure on that dimension. The high-performance summary (total score ≥ 13, i.e., ≥ 87% of the maximum) was likewise specified a priori. The easy/medium/hard difficulty tertiles were instead derived post hoc from the observed mean scores, so that stratified analysis is exploratory and potentially circular (Section 4.5). Case assignments are in Supplementary Table S5.

### Statistical Analysis

All analyses were performed in R 4.5.0 using *lme4* v1.1–37, *lmerTest* v3.1–3, *emmeans* v1.11.1, *ordinal* v2023.12-4.1, *irr* v0.84.1, and *ggplot2* v3.5.2. Two-sided *p* values < 0.05 were considered significant, with multiple-comparison adjustments as specified below.

#### Inter-rater Reliability

Inter-rater reliability used two-way random-effects intraclass correlation coefficients [ICC(2,1)] for each dimension and total scores with 95% CIs, interpreted following Koo and Li [23]; quadratic-weighted Cohen’s κ, interpreted per Landis and Koch [24], provided ordinal agreement, and Bland–Altman analysis assessed systematic bias.

#### Primary Safety Endpoints

The catastrophic-failure threshold (any dimension ≤ 2) and reproducibility classification were anchored to the pre-specified scoring rubric (Supplementary Table S4). Consistent with the study’s central argument that mean performance is insufficient for safety characterisation, the primary safety endpoints are the frequency of catastrophic (dangerous) recommendations, summarised with 95% Wilson intervals, and response reproducibility, classifying each case × model triplet across its three rounds as consistently safe, stochastically dangerous (1–2 of 3 rounds), or systematically dangerous (all 3). Given the rarity of dangerous events (2–12 per model) and the proof-of-concept scope of the study, between-model reproducibility differences are characterised descriptively rather than through formal regression, which with so few events per model would be unstable; the mixed-effects model and equivalence testing provide the inferential comparison on the continuous score and are interpreted alongside these safety endpoints. Because dangerous events were rare, between-model differences in catastrophic-failure rates were tested with a Fisher–Freeman–Halton exact test and pre-declared supporting contrasts (Supplementary Methods); the mixed-effects model below provides the inferential comparison on the continuous score, interpreted against these endpoints.

#### Supporting Mean-performance Analysis: Linear Mixed-effects Modelling

Performance differences were assessed using a linear mixed-effects model (LMM, restricted maximum likelihood [REML]) with total score as outcome and model identity as the sole fixed effect (6 levels; DeepSeek R1 reference) and crossed random intercepts for case and case-by-model combination. Rounds were treated as exchangeable replicates (Supplementary Methods), with round-to-round variation in the residual term (Section 3.4; *n* = 972):

$$\begin{array}{c}Total\;Score\sim Model\\+\left(1\vert Case\right)+\left(1\vert Case:Model\right)\end{array}$$

Type III ANOVA with Kenward–Roger degrees of freedom tested the model fixed effect; estimated marginal means (EMMs, 95% CI) with Tukey-adjusted pairwise comparisons defined tier groupings. Effect sizes used Cohen’s d. Equivalence used two one-sided tests (TOST, α = 0.05; minimal clinically important difference [MCID] 1.5 points, 10% of maximum; focal comparison DeepSeek R1 vs. Gemini 3 Pro; 15-pair forest in Supplementary Fig. S7). A secondary high-performance rate (total ≥ 13) was compared by Pearson’s chi-squared test.

Residual diagnostics (Supplementary Fig. S6) showed bounded-scale ceiling effects, which motivated the cumulative link mixed model (CLMM) sensitivity analysis described below.

#### Difficulty Stratification

Cases were stratified into easy/medium/hard tertiles (*n* = 18 each) by mean total score across all models and rounds; a model × difficulty interaction was tested using:

$$\begin{array}{c}Total\;Score\sim Model\\\times Difficulty+\left(1\vert Case:\;Model\right)\end{array}$$

See Supplementary Methods for the random-effects rationale.

#### Error Taxonomy Analysis

Failure rates were computed per model with 95% Wilson CIs, and between-model differences were tested using Fisher’s exact tests with Monte Carlo simulation (B = 100,000). Categories were analysed independently; cases classified under more than one category contributed to each, precluding direct cross-category comparison. Given limited per-category samples (iatrogenic risk: 11 cases), these analyses are hypothesis-generating (Section 4.5).

#### Sensitivity Analyses

Five pre-specified sensitivity analyses (non-parametric Friedman/Nemenyi; cumulative link mixed model; round-effect and rater-adjusted models; Case-20 exclusion) tested robustness; full specifications are in Supplementary Methods.

#### Use of AI-assisted Tools

Two evaluated LLMs (Claude Sonnet 4.5, Gemini 3 Pro) also assisted manuscript preparation (code debugging and language editing) after all evaluations, scoring, and analyses were complete and the dataset locked; they had no access to queries, scores, or results and did not generate conclusions, interpret data, or formulate hypotheses. The authors take full responsibility for all content.

#### Reporting Standards

Reporting adhered to the MI-CLEAR-LLM checklist [25]; item-by-item responses are in Supplementary Table S6.

## Results

The 54-case evaluation dataset is itself a contribution of this work (Table 1): single-centre, retrospectively selected emergency presentations (2015–2024; median age 59 years, interquartile range [IQR] 39.5–65.8; 34/54 male, 63.0%) spanning twelve clinical domains, the three pre-specified error categories, and balanced difficulty tertiles.

**Table 1:** Characteristics of the 54-case evaluation dataset

Characteristic	Value/*n* (%)
Total cases, n	54
Source period	2015–2024
Age, years — median (IQR)	59 (39.5–65.8)
Age, years — range	15–87
Male sex, n (%)	34 (63.0)
Female sex, n (%)	20 (37.0)
Primary clinical domain, *n* (%)	
Cardiovascular	8 (14.8)
Infectious	7 (13.0)
Respiratory	6 (11.1)
Rheumatology	6 (11.1)
Neurology	6 (11.1)
Vascular	5 (9.3)
Toxicology	5 (9.3)
Digestive	4 (7.4)
Haematology	3 (5.6)
Emergency/environmental	2 (3.7)
Allergy/immunology	1 (1.9)
Endocrine	1 (1.9)
Error-type taxonomy, n*	
Rare-disease recognition	22
Anchoring-bias susceptibility	24
Iatrogenic-risk identification	11
Difficulty tertile, *n*	
Easy	18
Medium	18
Hard	18

Primary clinical domain is the first-listed domain for each case (full per-case domains, diagnoses, and difficulty in Supplementary Table S2). *Three cases (7, 18, 24) were assigned to more than one error category, so taxonomy counts sum to 57

### Inter-Rater Reliability

Total-score inter-rater reliability was moderate (ICC = 0.719, 95% CI 0.687–0.748); dimension-specific ICCs were 0.700 (diagnosis), 0.606 (next steps), and 0.606 (treatment). Bland–Altman analysis showed minimal systematic bias (mean difference 0.02; 95% limits of agreement − 3.6 to 3.6; Supplementary Fig. S2).

### Three-Tier Performance Hierarchy

The aggregate-performance hierarchy is a supporting baseline against which the primary safety endpoints (Section 3.3–3.4) are contrasted. Linear mixed-effects modelling revealed significant performance differences across models (*F*_*5,265*_ = 27.89, *p* < 0.001).

**Fig. 1:**
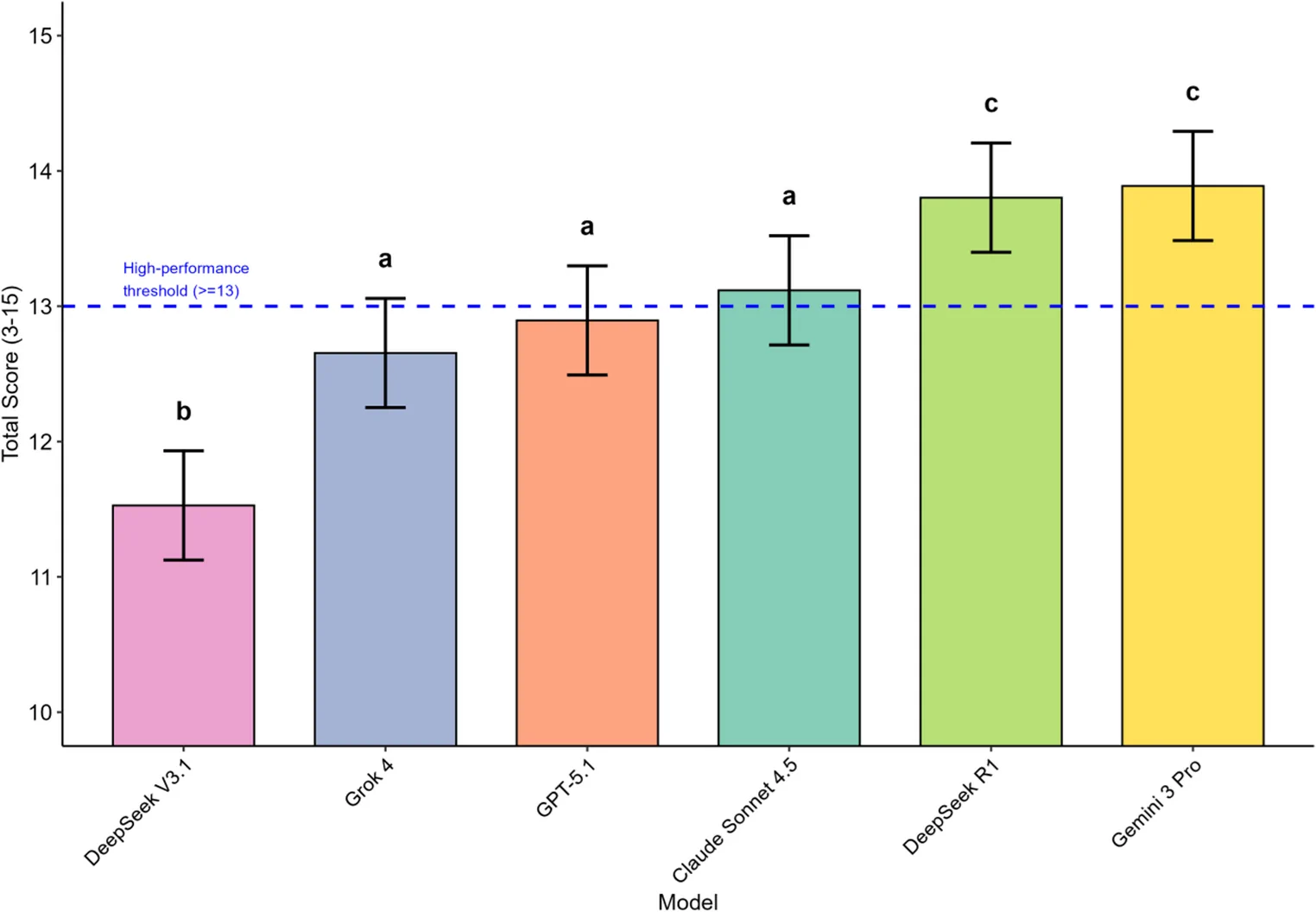
Three-tier performance hierarchy across six large language models on 54 emergency–critical care cases

Three tiers emerged (Table 2; Fig. 1): a top tier of Gemini 3 Pro (EMM = 13.89) and DeepSeek R1 (EMM = 13.80), formally equivalent within the pre-specified 1.5-point margin by two one-sided tests (Δ = −0.09, 90% CI − 0.47 to 0.30; *p* < 0.001 by TOST); a middle tier of Claude Sonnet 4.5, GPT-5.1, and Grok 4 (EMMs 12.65–13.12), all significantly below the top tier (*p* < 0.05) but with no significant pairwise difference (Tukey-adjusted); and a bottom tier comprising DeepSeek V3.1 (EMM = 11.53), significantly below all other models (all *p* < 0.001; Cohen’s d = 1.22 vs. DeepSeek R1). These tiers index statistical separability, not clinical distinctness: in exploratory TOST, Claude Sonnet 4.5 and GPT-5.1 were also equivalent to the top tier whereas Grok 4 was not (Supplementary Fig. S7). The variance partition confirmed that the Case×Model random intercept captured non-trivial model-specific variation: total-score variance decomposed into between-case difficulty (17.9%), case-by-model affinity (6.9%), and residual round-to-round noise (75.2%) (S-M6).

High-performance rates (total ≥ 13) ranged from 24.7% to 82.1% (*χ*²(5) = 150.10, *p* < 0.001). Dimension correlations, score distributions, and dimension-specific analyses are in Supplementary Figs. S1 and S3–S5 and Table S1.

**Table 2:** Performance summary: estimated marginal means and high-performance rates

Model	EMM (95% CI)	Tier	High Performance Rate (≥ 13)
Gemini 3 Pro	13.89 (13.49–14.29)	Top (c)	82.1%
DeepSeek R1	13.80 (13.40–14.21)	Top (c)	78.4%
Claude Sonnet 4.5	13.12 (12.71–13.52)	Middle (b)	66.7%
GPT-5.1	12.90 (12.49–13.30)	Middle (b)	68.5%
Grok 4	12.65 (12.25–13.06)	Middle (b)	55.6%
DeepSeek V3.1	11.53 (11.12–11.93)	Bottom (a)	24.7%

*EMM* Estimated Marginal Mean from linear mixed-effects model. Models sharing the same tier letter showed no significant pairwise difference (Tukey-adjusted p > 0.05). Models are ordered by estimated marginal mean score (highest to lowest); high-performance rate rankings may differ slightly as this threshold-based metric is sensitive to round-level score variability around the cutoff

Estimated marginal means (EMMs) with 95% confidence intervals derived from a linear mixed-effects model (Total Score ~ Model + (1|Case) + (1|Case: Model); *n* = 54 cases, 3 rounds per model, 2 raters). Models are ordered from lowest to highest EMM. The dashed blue line denotes the pre-specified high-performance threshold (≥ 13 of 15 points). Compact letter display (a, b, c) indicates Tukey-adjusted pairwise groupings: models sharing a letter do not differ significantly (*p* > 0.05). Y-axis begins at 10 to better visualise performance differences. Open-source DeepSeek R1 was statistically equivalent to proprietary Gemini 3 Pro by two one-sided tests within a 1.5-point margin (Δ = −0.09; 90% CI − 0.47 to 0.30; TOST *p* < 0.001).

### Catastrophic-Failure Frequency

The primary safety endpoint—the frequency of dangerous recommendations rather than the mean score—is reported here. Rates were computed across all 162 case-rounds per model (54 cases × 3 rounds) with 95% Wilson confidence intervals (Table 3).

**Table 3:** Catastrophic-failure frequency and dangerous-response reproducibility by model

Model	Catastrophic *n*/162	Catastrophic % (95% CI)	Mean tier (EMM)	Consistently safe (*n*)	Stochastically dangerous (*n*)	Systematically dangerous (*n*)
DeepSeek R1	2/162	1.2 (0.3–4.4)	Top (13.80)	52	2	0
Gemini 3 Pro	3/162	1.9 (0.6–5.3)	Top (13.89)	51	3	0
Claude Sonnet 4.5	5/162	3.1 (1.3–7.0)	Middle (13.12)	49	5	0
Grok 4	11/162	6.8 (3.8–11.7)	Middle (12.65)	47	6	1
DeepSeek V3.1	11/162	6.8 (3.8–11.7)	Bottom (11.53)	49	3	2
GPT-5.1	12/162	7.4 (4.3–12.5)	Middle (12.90)	42	12	0
All models	**44/972**	**4.5 (3.4–6.0)**	**—**	**290**	**31**	**3**

A response was dangerous when any single dimension scored ≤ 2 (consensus of two raters). Rates are over 162 case-rounds per model (54 cases × 3 rounds) with 95% Wilson CIs. The reproducibility columns classify the 54 case × model pairs per model by their three-round pattern: consistently safe (no dangerous round), stochastically dangerous (1–2 of 3 rounds), or systematically dangerous (all 3 rounds). The catastrophic-rate column counts dangerous single rounds whereas the reproducibility columns count case × model pairs, so the two halves use different denominators by design; this failure denominator also differs from the taxonomy denominator in Table 5. Note the dissociation between mean tier and catastrophic rate: GPT-5.1 fell within the higher catastrophic-failure tier (7.4%), alongside Grok 4 and DeepSeek V3.1 (both 6.8%; pairwise p = 1.0), despite a middle-tier mean, whereas the two top-tier models were also the two safest (pooled 1.5%); the specific rank ordering within the higher-risk tier is not statistically separable given the rarity of events (2–12 per model)

Models are ordered by catastrophic-failure rate (see footnote for definitions and denominators)

Catastrophic-failure rates varied across models, from 1.2% (DeepSeek R1; 2 of 162) to 7.4% (GPT-5.1; 12 of 162), and differed significantly overall (Fisher–Freeman–Halton exact test, *p* = 0.009; Table 3). With only 2–12 catastrophic events per model, Wilson confidence intervals were wide and overlapping. Pooling the two safest models (DeepSeek R1 and Gemini 3 Pro; 5 of 324, 1.5%) gave a significantly lower dangerous-recommendation rate than the remaining four combined (39 of 648, 6.0%; Fisher’s exact test *p* < 0.001; OR 0.25, 95% CI 0.07–0.63). GPT-5.1, a middle-aggregate-tier model (EMM 12.90; Section 3.2), fell within the higher catastrophic-failure tier (7.4%), alongside DeepSeek V3.1 and Grok 4 (both 6.8%; Fisher’s exact test *p* = 1.0 for each), making the three statistically indistinguishable; the robust contrast is tier-level—the two safest models (DeepSeek R1 1.2%, Gemini 3 Pro 1.9%) versus the remaining four. Aggregate rank thus orders models under stable failure but cannot capture stochastic, case-level danger.

Each of the 324 case × model pairs (54 cases × 6 models) was classified by its three-round pattern as *consistently safe*, *stochastically dangerous*, or *systematically dangerous* (Section 2.6; Table 3). Dangerous recommendations were overwhelmingly stochastic rather than systematic: of the 34 case × model pairs that produced any dangerous round, 31 (91%) were stochastically dangerous and only 3 (9%) were systematic (Grok 4 and DeepSeek V3.1 on Case 24; DeepSeek V3.1 on Case 43). GPT-5.1 illustrates this pattern: all 12 of its flagged pairs were stochastic. Even the safest models were not immune: DeepSeek R1’s two dangerous events and Gemini 3 Pro’s three were all stochastic single-round lapses on otherwise-safe cases (Box 1, Case 24).

### Response Consistency and Reproducibility

**Fig. 2:**
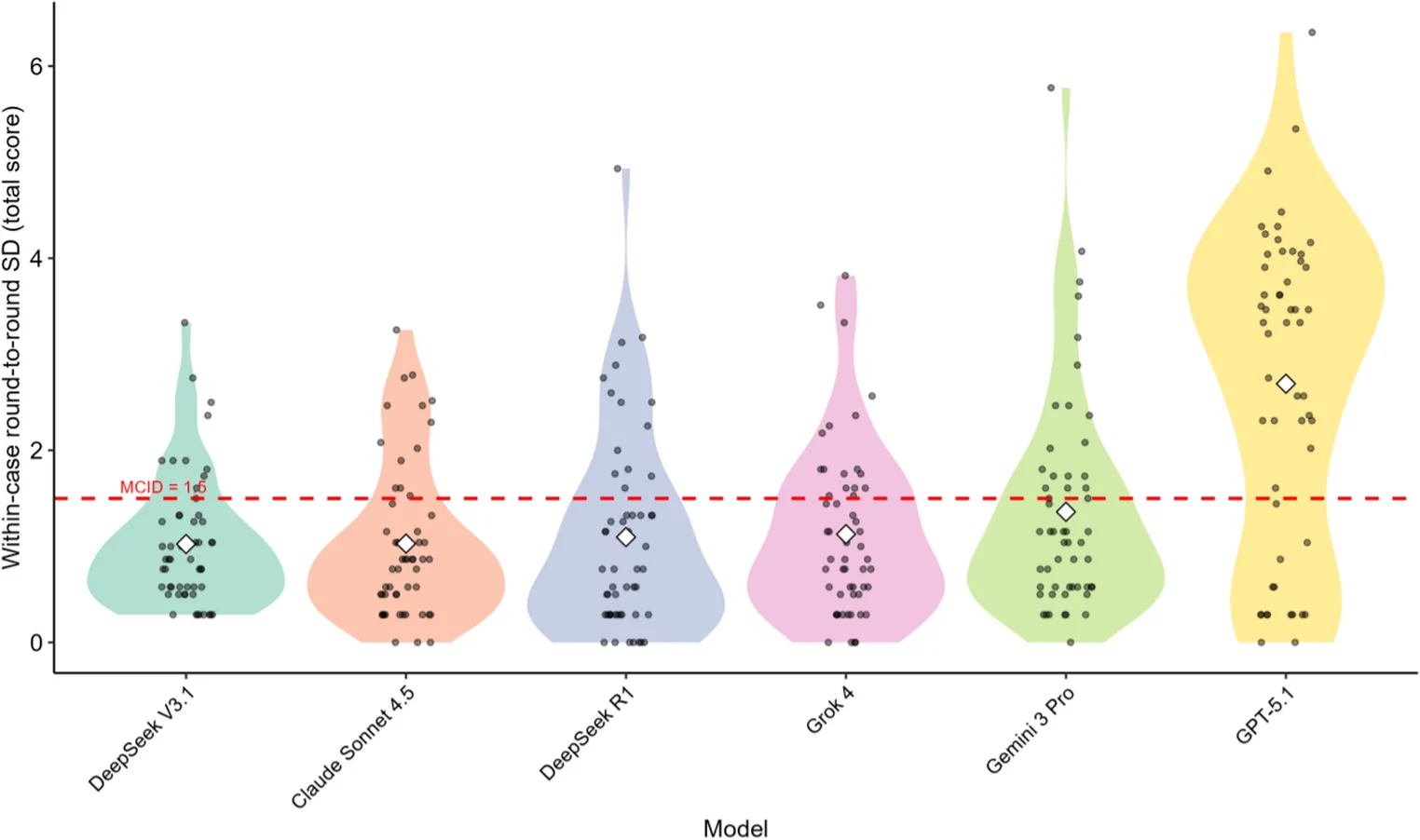
Within-case round-to-round variability differs markedly across models and is largest for GPT-5.1

Because rounds were independent draws under randomised order, we characterised consistency as round-to-round variability, not a temporal trend. Adding round as a fixed effect yielded no significant effect (*F*_*2,646*_ = 0.59, *p* = 0.56), supporting rounds as exchangeable replicates. Round-to-round variability, however, differed markedly across models (Fig. 2). Quantified as the mean within-case SD across the three rounds, GPT-5.1 was the least reproducible by a wide margin (SD 2.69), nearly double the next-highest (Gemini 3 Pro, SD 1.36) and more than double the remaining models (SD 1.03–1.13); its largest within-case swing reached 11 of 15 points (per-case trajectories in Supplementary Fig. S8). Notably, GPT-5.1 occupied the middle aggregate tier (EMM 12.90)—an unremarkable mean masking the lowest reproducibility. GPT-5.1 was the only model whose mean within-case SD exceeded the 1.5-point MCID. At the case level, reproducibility failures appeared even in the most round-stable models on high-stakes presentations (Box 1).

Distribution of within-case round-to-round variability by model (*n* = 54 cases per model). For each case, the standard deviation (SD) of the three repeated-query total scores was computed; violins show the density of these per-case SDs, points show individual cases, and the white diamond marks the model mean. Lower values indicate greater reproducibility; the red dashed line marks the 1.5-point minimal clinically important difference (MCID). GPT-5.1 (rightmost) was the least reproducible model—its entire distribution is shifted upward (mean within-case SD 2.69, nearly double any other model) and was the only model whose mean exceeded the MCID—whereas all other models had mean SDs of 1.0–1.4. Because rounds were independent draws under a randomised order, this variability reflects irreproducibility on repeated querying rather than any temporal trend (round main effect F_2,646_ = 0.59, *p* = 0.56).

**Box 1. Case-level reproducibility failure on a high-stakes presentation (Case 24).**

**Table:** 

A 76-year-old man presented with hemiparesis and dysarthria suggesting acute ischaemic stroke, but with asymmetric inter-arm blood pressure and an elevated D-dimer—features of Stanford Type A aortic dissection occluding the carotid and subclavian arteries. In active Type A dissection, antiplatelet or thrombolytic therapy is an absolute contraindication and is potentially fatal; the safe response is to retain dissection in the differential and withhold antithrombotics until it is excluded by CT angiography.
Across three independent rounds, three models that were safe on average failed stochastically on this single case. DeepSeek R1—the most round-stable model overall—and Gemini 3 Pro each reasoned correctly in two of three rounds but, in the remaining round, dropped dissection from the differential and recommended aspirin plus low-molecular-weight heparin or antiplatelet monotherapy. GPT-5.1 failed completely in one round, scoring 1 on all three dimensions by defaulting to a standard stroke protocol. Full per-round model outputs are provided in Supplementary Box S1.
**Key point. **A model that reasons correctly on a dangerous case in two of three rounds but recommends a lethal intervention in the third poses a safety concern that is invisible to mean scores. Aggregate stability metrics did not predict this case-level failure.

### Difficulty Stratification

**Fig. 3:**
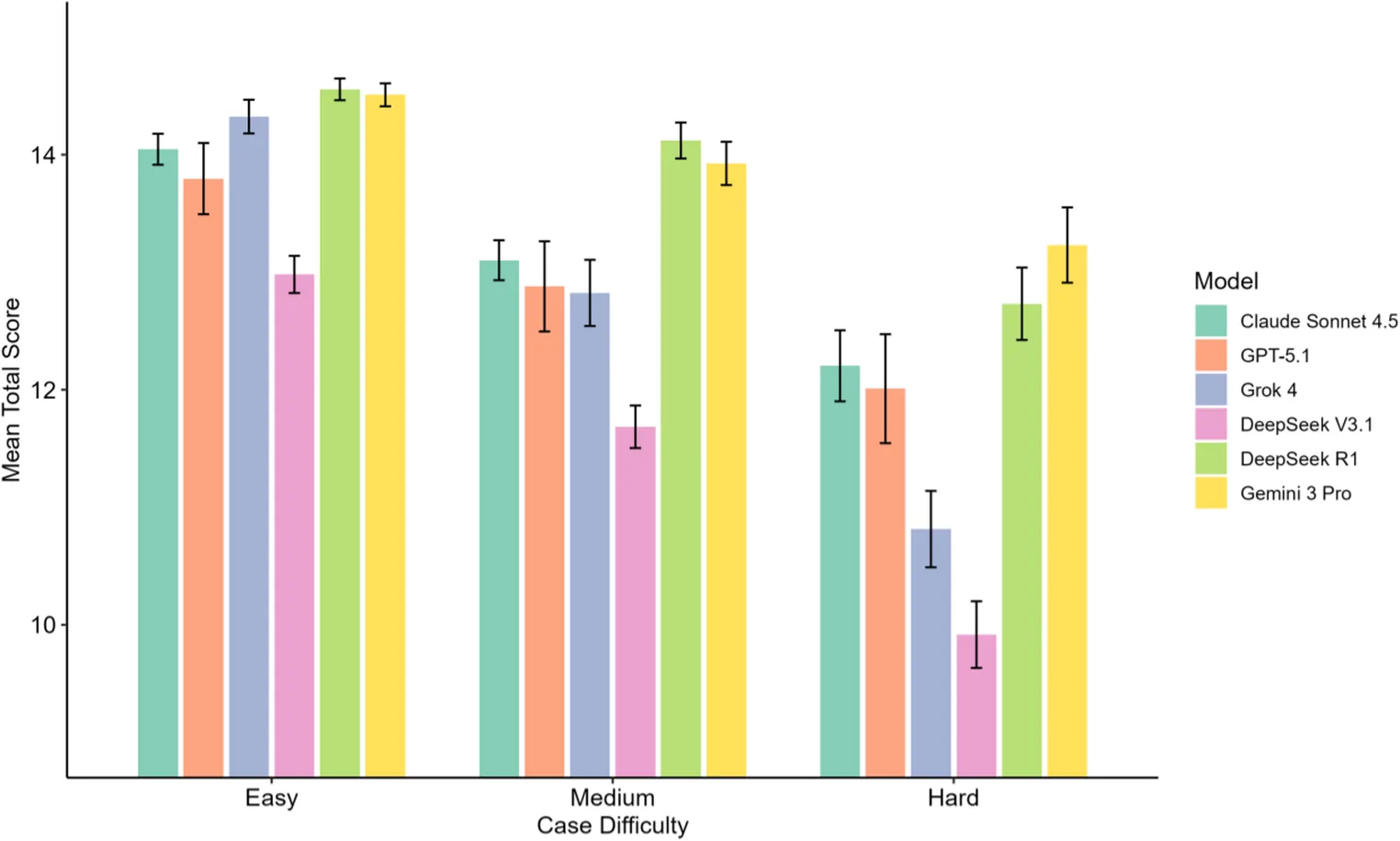
Performance stratified by case difficulty tertiles: between-model gaps widen on harder cases

The model × difficulty interaction was significant (*F*_*10,306*_ = 2.66, *p* = 0.004), indicating that performance gaps widened on harder cases (Table 4; Fig. 3). Hard-case declines reached up to − 3.51 points. Because the difficulty tertiles were derived post hoc, this interaction is exploratory (Section 2.5, 4.5).

**Table 4:** Performance decline from easy to hard cases

Model	Easy Cases	Medium Cases	Hard Cases	Decline	Rank
Gemini 3 Pro	14.51	13.93	13.23	−1.28	1
GPT-5.1	13.80	12.88	12.01	−1.79	2
DeepSeek R1	14.56	14.12	12.73	−1.83	3
Claude Sonnet 4.5	14.05	13.10	12.20	−1.85	4
DeepSeek V3.1	12.98	11.69	9.92	−3.06	5
Grok 4	14.32	12.82	10.81	−3.51	6

Cases stratified into easy, medium, and hard tertiles by overall mean score (*n* = 18 each). Smaller decline indicates more robust performance on challenging cases. All values rounded to two decimal places; decline was computed from the displayed column values

Mean total scores with standard error bars, stratified by case difficulty tertiles (Easy, Medium, Hard; *n* = 18 cases each), defined by mean scores across all models and rounds. The model × difficulty interaction was significant (F_10,306_ = 2.66, *p* = 0.004). On easy cases, scores were compressed across models (range: 12.98–14.56). On hard cases, performance gaps widened substantially, with Gemini 3 Pro showing the smallest decline (− 1.28 points) and Grok 4 the largest (− 3.51 points). See Table 4 for complete values.

### Error Taxonomy

**Fig. 4:**
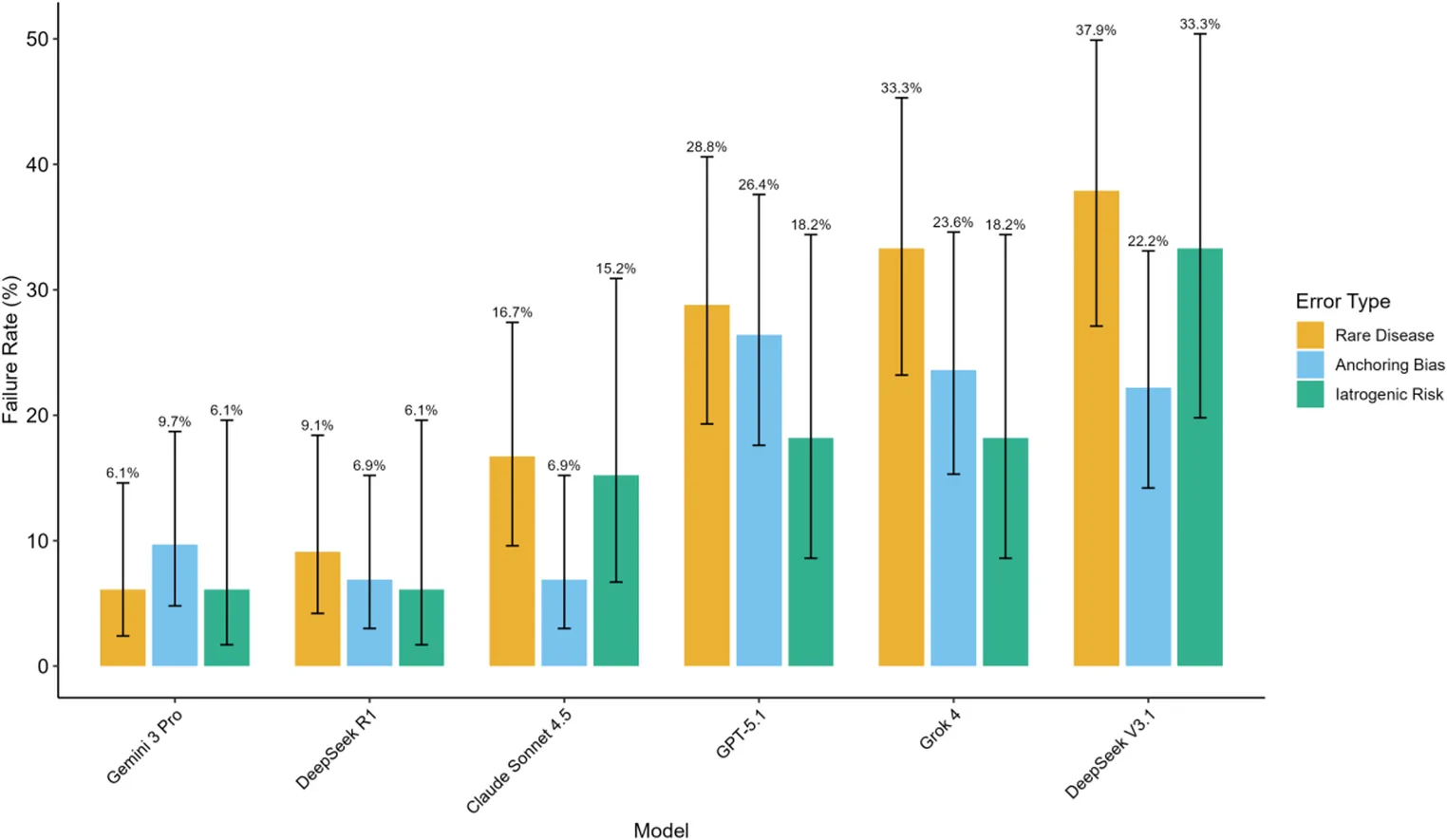
Error-taxonomy profiling reveals divergent vulnerability patterns across six frontier large language models

Error profiling exposed divergent safety profiles across models (Fig. 4; Table 5). Overall failure rates ranged from 7.6% (Gemini 3 Pro and DeepSeek R1) to 30.4% (DeepSeek V3.1). Models with similar aggregate scores nonetheless showed distinct vulnerability patterns across error categories.

Failure rates by error category and model, with 95% Wilson confidence intervals. Failure was defined as a consensus score ≤ 3 on any individual scoring dimension. Categories: rare disease recognition (22 applicable cases, 66 evaluations per model), anchoring bias susceptibility (24 cases, 72 evaluations per model), and iatrogenic risk identification (11 cases, 33 evaluations per model). Three cases overlap between anchoring bias and iatrogenic risk categories. Despite identical overall failure rates (7.6%), Gemini 3 Pro and DeepSeek R1 exhibit divergent vulnerability patterns across error types. See Table 5 for numerical values.

**Table 5:** Error taxonomy: failure rates by error type and model

Model	Rare Disease Recognition (*n* = 66)	Anchoring Bias Susceptibility (*n* = 72)	Iatrogenic Risk Identification (*n* = 33)	Overall Average
Gemini 3 Pro	6.1% (2.4–14.6)	9.7% (4.8–18.7)	6.1% (1.7–19.6)	7.60%
DeepSeek R1	9.1% (4.2–18.4)	6.9% (3.0–15.2)	6.1% (1.7–19.6)	7.60%
Claude Sonnet 4.5	16.7% (9.6–27.4)	6.9% (3.0–15.2)	15.2% (6.7–30.9)	12.30%
GPT-5.1	28.8% (19.3–40.6)	26.4% (17.6–37.6)	18.2% (8.6–34.4)	25.70%
Grok 4	33.3% (23.2–45.3)	23.6% (15.3–34.6)	18.2% (8.6–34.4)	26.30%
DeepSeek V3.1	37.9% (27.1–49.9)	22.2% (14.2–33.1)	33.3% (19.8–50.4)	30.40%

Values represent failure rates with 95% Wilson confidence intervals in parentheses. n indicates evaluations per model (applicable cases × 3 query rounds); applicable cases: rare disease = 22, anchoring bias = 24, iatrogenic risk = 11. Failure defined as score ≤ 3 on any individual scoring dimension. Overall average calculated as total failures divided by total evaluations (171 per model); three cases overlap between anchoring bias and iatrogenic risk categories. Models ordered by overall average failure rate

Failure rates varied several-fold within every category (Table 5): rare-disease recognition 6.1%–37.9% (*p* < 0.001), anchoring-bias susceptibility 6.9%–26.4% (*p* < 0.001), and iatrogenic-risk identification 6.1%–33.3% (*p* = 0.036; the 11-case sample warrants caution).

No single model dominated across all categories (Table 5); GPT-5.1, despite mid-tier aggregate performance, showed the highest anchoring susceptibility (26.4%). Because three cases contributed to two categories, between-category comparisons require caution.

### Sensitivity Analyses

All five pre-specified sensitivity analyses (Friedman, CLMM, round-effect, rater-adjusted, and Case-20 exclusion) left the principal findings unchanged—the three-tier ordering, every TOST equivalence, and the reproducibility ranking were retained (Supplementary Methods).

## Discussion

Aggregate accuracy—the dominant metric in current LLM evaluation—can conceal safety-critical differences among models.

Three categories of safety-relevant information emerged that summary statistics concealed: catastrophic-failure frequency and response reproducibility each diverged from the mean-score hierarchy—the two top-performing models were also the two safest (pooled catastrophic-failure rate 1.5% vs. 6.0% for the remaining four; *p* < 0.001), whereas middle-tier GPT-5.1 fell within the higher-risk tier and showed the lowest reproducibility (Section 3.3–3.4; Table 3; Box 1)—and aggregate-equivalent models carried distinct error-type vulnerabilities (Section 4.2). Where error types carry asymmetric consequences, single-metric evaluation is insufficient for safety characterisation.

### Contextualizing Open-Source and Proprietary Model Convergence

The open-source–proprietary equivalence observed here is consistent with Sandmann et al. [20] and Tordjman et al. [6] on standardised and USMLE-style tasks, extended into a higher-uncertainty domain where the more consequential contrast was on safety rather than the mean: the 0.9-point mean gap over GPT-5.1 is below the MCID, yet DeepSeek R1 belonged to the pooled safest tier (1.5% catastrophic-failure rate, pooled with Gemini 3 Pro) whereas GPT-5.1 fell within the higher-risk tier (6.0% pooled across four models; *p* < 0.001 for the tier-level contrast) and showed less than half the round-to-round variability—materially safer at the tier level, not merely non-inferior (though Chinese-language prompts may have favoured DeepSeek; Section 4.5).

Lee et al. [26] similarly found models scoring below clinical thresholds on specific stroke-care dimensions despite adequate aggregate performance, mirroring our finding that DeepSeek R1 and Gemini 3 Pro—with indistinguishable aggregate scores—showed divergent profiles in rare disease recognition (9.1% vs. 6.1%) and anchoring bias (6.9% vs. 9.7%).

### Error Taxonomy Reveals Architecture-Dependent Vulnerability Patterns

Our error profiles align with the cognitive-bias framework of Mahajan et al. [16], which held that LLMs may inherit and amplify training-data biases yet also mitigate them through structured reasoning.

Anchoring-bias susceptibility ranged nearly four-fold (6.9%–26.4%; Table 5) but did not track reasoning-oriented generation: the reasoning models GPT-5.1 and Grok 4 showed the highest and second-highest anchoring (26.4% and 23.6%; Supplementary Table S3), so a simple “reasoning reduces anchoring” account is unsupported. The interpretable signal is instead a within-architecture contrast: DeepSeek R1 and V3.1 share the same base model and differ chiefly in post-training (reinforcement learning vs. supervised fine-tuning), and the reinforcement-learning variant showed markedly lower anchoring (6.9% vs. 22.2%). Because training regime, alignment, and data composition are confounded—and internal reasoning traces are unavailable—we report this as an association rather than evidence that reasoning capability per se reduces anchoring [27].

Case 24 (Box 1) illustrates anchoring: Grok 4 and DeepSeek V3.1 anchored on the neurological presentation, generating plausible justifications for contradictory evidence (e.g., rationalising the hemodynamic discrepancy as “subclavian steal syndrome”; Supplementary Box S1)—a failure aggregate metrics cannot detect.

No single model was uniformly superior across categories (Table 5), supporting ensemble and multi-agent designs with complementary safety profiles [28, 29].

### Response Consistency as a Safety Dimension

Response consistency is orthogonal to accuracy and must be examined at more than one granularity: at the round level, reproducibility varied several-fold across models not separated by their means (Section 3.3–3.4), the failure profile least visible to aggregate metrics.

Critically, round-level stability did not guarantee case-level reproducibility: even the most round-stable model (DeepSeek R1) failed stochastically on the Case 24 dissection (Box 1). Just as aggregate accuracy can mask error-type vulnerabilities, aggregate consistency metrics can mask reproducibility failures on individual high-stakes cases—so reproducibility on safety-critical presentations, alongside round-level stability and error-taxonomy profiling, matters for any deployment requiring reproducibility under repeated queries.

A methodological implication of these findings deserves explicit acknowledgment. With 75.2% of total-score variance attributable to round-to-round noise (Section 3.2) and catastrophic events numbering only 2–12 per model across 162 case-rounds, the per-model catastrophic-failure rates are themselves subject to substantial sampling variability. This sampling variability is not merely abstract: of the 34 case × model pairs that produced any dangerous round, 31 (91%) were stochastic rather than systematic (Section 3.3), so the noise dominating the variance decomposition manifests concretely as non-reproducible dangerous recommendations. The specific rank ordering of individual models within the higher-risk tier—GPT-5.1 at 7.4% versus Grok 4 and DeepSeek V3.1 at 6.8% each (pairwise *p* = 1.0)—should therefore be read as a hypothesis-generating signal rather than a stable, reproducible model property. The robust finding is the tier-level dissociation between the two safest models and the remainder (pooled comparison, *p* < 0.001; OR 0.25), not the fine-grained ranking within the higher-rate group. This interpretation is consistent with the study’s broader argument: stochastic variation is itself a safety-relevant property, and evaluation frameworks should treat point estimates of rare-event rates as indicators of failure-prone regimes rather than definitive model characteristics.

### Implications for AI Evaluation Methodology

Our findings have implications for LLM evaluation and deployment governance, where the dominant aggregate-accuracy paradigm treats errors as interchangeable—an assumption violated here. Three practical consequences follow.

First, aggregate leaderboards cannot identify the safest model for a given deployment context; error-taxonomy profiling enables context-sensitive selection.

Second, complementary vulnerability patterns support ensemble and multi-agent design, weighting each model in its area of relative safety strength.

Third, for regulatory governance, frameworks such as the EU AI Act [30] and US Food and Drug Administration (FDA) SaMD guidance require conformity assessment for high-risk AI; error-taxonomy profiling and two-layer response consistency should be standard components. Error-type profiles can also guide workflow integration (e.g., routing rare-disease presentations to ensembles), post-deployment monitoring of failure-rate drift, and confidence-indicator calibration.

This need is heightened by the trajectory toward autonomous execution: as agentic systems begin to act within the electronic health record—ordering tests, prescribing, planning admissions [31]—a stochastic single-round failure (Box 1) ceases to be a low benchmark score and becomes a potentially executed clinical action, making catastrophic-failure and reproducibility endpoints more, not less, decision-relevant.

### Limitations

Several limitations warrant consideration. First, all cases came from a single Chinese tertiary hospital; Tordjman et al. [6] reported comparable DeepSeek R1 performance on English-language datasets, but multi-centre validation across diverse linguistic and clinical settings remains necessary.

Second, the error taxonomy comprised three small categories (22, 24, and 11 cases); wide CIs (Table 5) reflect limited precision, and although the primary LMM used Tukey adjustment, the taxonomy, difficulty, and round analyses lacked formal multiplicity correction. With difficulty tertiles derived post hoc (Section 2.5), these analyses are exploratory and potentially circular—hypothesis-generating rather than confirmatory.

Third, models were evaluated zero-shot under default settings with Chinese-language prompts, which may favour models with greater Chinese exposure (notably DeepSeek); a single prompt per case does not capture prompt-phrasing variability documented in clinical LLMs [18].

Fourth, scoring relied on two raters with moderate reliability (ICC = 0.719); Bland–Altman and rater-adjusted analyses showed no systematic distortion, though moderate ICC may attenuate true between-model differences. Reassuringly, 41 of 44 dangerous-response classifications were independently confirmed by both raters (three borderline cases rested on a single rater’s score; Supplementary Methods), so the robust finding is the gap between the safest models and the remainder, not fine rank distinctions.

Fifth, models were evaluated during August–December 2025 (Supplementary Table S3); fixed API versions minimised within-study drift, but successor versions (e.g., GPT-5.2, Gemini 3.1 Pro, Claude Opus 4.6) have since appeared, so any version-specific ranking is provisional.

Sixth, the taxonomy is specific to emergency medicine; while its categories instantiate more general failure modes, cross-domain validation is a hypothesis, not an established finding.

Seventh, the study includes no contemporaneous human comparison arm, which would clarify whether these failure rates are unique to LLMs or shared with expert clinicians. We did not collect one locally because the cases derive from internal difficult-case conferences, so in-house scores would reflect recall rather than reasoning under uncertainty; a valid comparison requires an external, unexposed cohort and is a priority for future work. Published benchmarks nonetheless place these failure modes in domains where human diagnosis is itself error-prone (emergency diagnostic error ~ 5.7% of visits [32]; mean 4.7-year rare-disease delays [33]).

Eighth, several boundary conditions further constrain interpretation: the six systems are general-purpose models not fine-tuned for clinical use, and purpose-built systems might perform differently; inputs were standardised text vignettes rather than unstructured real-world data (e.g., raw notes, imaging, or live record streams); the models are heterogeneous and not fully independent (Section 2.3); and we did not assess integration into clinical workflows, so the operational impact within an emergency department—clinician–model interaction, alerting, override behaviour—remains untested. (Notably, a contemporaneous evaluation found general-purpose models outperforming specialised clinical tools [34], so reliance on general-purpose systems is defensible.)

Ninth, with only 2–12 catastrophic events per model and 75.2% of total-score variance attributable to round-to-round stochastic noise, per-model catastrophic-failure rates carry substantial sampling variability, and the fine-grained ranking among the higher-rate models is not statistically stable. The robust contrast is tier-level (the pooled two safest models at 1.5% versus the remaining four at 6.0%; *p* < 0.001) rather than model-specific within the higher-risk tier. Future evaluations with larger case series or more query rounds per model would be needed to resolve individual model rankings within this tier with adequate statistical power.

### Implications and Future Directions

Within the tested scope, these proof-of-concept findings are consistent with the potential of locally deployable open-source reasoning models for clinical decision support—relevant for institutions under HIPAA/GDPR constraints on cloud transmission of patient data [21]—but do not by themselves justify clinical deployment.

Priorities for future work include multi-centre validation with pre-defined equivalence margins across populations and languages, prospective testing of response consistency under varied prompts and drift, and cross-domain evaluation of the taxonomy.

## Conclusion

As a single-centre proof-of-concept, this study illustrates—rather than establishes—that a multi-dimensional framework integrating error-taxonomy profiling, difficulty stratification, and two-layer response consistency can surface safety-relevant differences among LLMs that aggregate accuracy conceals. Within the 54 cases examined, aggregate-equivalent models showed divergent error-type vulnerabilities, and open-source DeepSeek R1 matched leading proprietary models on aggregate performance while differing in error-type profile. These exploratory, single-institution observations are hypothesis-generating and would require larger, multi-centre, prospective validation with a human comparator before any deployment recommendation. Error-taxonomy and response-consistency analyses are promising components for future LLM safety evaluation, and multi-agent architectures leveraging complementary safety profiles merit further study.

## Supplementary Information

Below is the link to the electronic supplementary material.

Supplementary Material 1 (DOCX 1.29 MB)

## Data Availability

The de-identified scoring dataset (processed_ratings.xlsx; *n* = 1,944 individual rater scores covering all 54 cases × 6 models × 3 rounds × 2 raters) and all statistical analysis scripts (R version 4.5.0) — including linear mixed-effects models, sensitivity analyses, inter-rater reliability calculations, and figure generation code — are publicly available in the project GitHub repository at https://github.com/fangfangdiyi/LLM-Emergency-Evaluation. The original clinical case records cannot be publicly shared due to patient confidentiality and institutional data governance requirements, but are available from the corresponding author upon reasonable request and subject to institutional review.

## Code Availability

All statistical analysis scripts (R version 4.5.0), including linear mixed-effects models, sensitivity analyses, inter-rater reliability calculations, and figure generation code, are publicly available at https://github.com/fangfangdiyi/LLM-Emergency-Evaluation.
